# Improving the Photostability and Antioxidant Activity of Resveratrol via Incorporation in Two Types of Polymeric Nanoparticles

**DOI:** 10.3390/ijms27135846

**Published:** 2026-06-29

**Authors:** Lyubomira Radeva, Miroslava Demireva, Aleksandar Belchev, Yordan Yordanov, Ivanka Spassova, Daniela Kovacheva, Virginia Tzankova, Krassimira Yoncheva

**Affiliations:** 1 Faculty of Pharmacy, Medical University of Sofia, 1000 Sofia, Bulgaria; l.radeva@pharmfac.mu-sofia.bg (L.R.); miroslava_demireva@abv.bg (M.D.); ale.belchev@gmail.com (A.B.); yyordanov@pharmfac.mu-sofia.bg (Y.Y.); vtzankova@pharmfac.mu-sofia.bg (V.T.); 2 Institute of General and Inorganic Chemistry, Bulgarian Academy of Sciences, 1113 Sofia, Bulgaria; ispasova@svr.igic.bas.bg (I.S.); didka@svr.igic.bas.bg (D.K.)

**Keywords:** resveratrol, Pluronic, albumin, micelles, nanospheres, photostability, antioxidant activity

## Abstract

The natural stilbene resveratrol is a widely researched molecule, owing to its antioxidant activity and abundance of pharmacological effects. However, its application is still hindered due to its low aqueous solubility, bioavailability and photostability. Therefore, in the current study, resveratrol was loaded in two types of polymeric nanoparticles, namely mixed Pluronic F127 and P123 micelles, and bovine serum albumin nanospheres. The loaded micelles and nanospheres possessed mean diameters of 33 nm and 145 nm, zeta potentials of −4 mV and −22.6 mV, and encapsulation efficiency levels of 89.4% and 76.2%, respectively. The aqueous solubility of resveratrol increased after loading, especially in the albumin nanospheres. A sustained release was observed, more pronounced for the micelles. The photostability of the encapsulated and pure resveratrol was evaluated under daylight exposure and UV irradiation. The micelles showed superior protective effect compared to the nanospheres. The antioxidant potentials of the formulations were examined through ABTS radical scavenging activity assay and in vitro cell model of oxidative stress in L929 fibroblasts. The resveratrol-loaded albumin nanospheres showed a more enhanced antioxidant effect. Thus, the encapsulation of resveratrol in both types of nanoparticles could be considered an advantageous approach due to improvements in solubility, stability and antioxidant activity.

## 1. Introduction

Resveratrol is a polyphenol whose structure consists of two phenol rings linked to each other by an ethylene bridge [1]. Its chemical structure (3, 5, 4′-trihydroxystilbene) exists in two isomeric forms: cis- and trans-resveratrol. The trans form has higher pharmacological activity. It possesses antioxidant activity that contributes to anti-inflammatory, cardioprotective, neuroprotective, and other effects [2,3]. The antioxidant activity of resveratrol strongly correlates with the redox characteristics of the phenolic -OH groups in the structure and the possibility for electron delocalization. The main mechanisms of this activity are direct (via inhibition of reactive oxygen species, which is mediated by NADPH oxidase) and indirect (via activation of antioxidant enzymes such as catalase, superoxide dismutase, and others, or by mitigating important signaling pathways) [3,4]. Plenty of studies show that resveratrol has significant therapeutic potential in diseases caused by oxidative stress. For instance, its ability to increase the expression of heme oxygenase 1 (HO-1) is a prerequisite for its use in the treatment of atherosclerosis; its ability to activate the adenosine 5′-monophosphate-activated protein kinase signaling pathway suggests that it has the potential to improve myocardial ischemia–reperfusion injuries in diabetic patients; and its activation of sirtuin 1 (SIRT1) participates in the promotion of wound healing [4].

Despite the various pharmacological activities of resveratrol, its unfavorable pharmacokinetic properties can lower therapeutic efficacy and impede its clinical application. For instance, its beneficial effects are hindered by its low bioavailability [1,5]. Taken orally, its absorption in humans is approx. 75% and, due to extensive intestinal and liver metabolism, the bioavailability is less than 1% [5]. Another significant issue of resveratrol is its low water solubility (approximately 0.05 mg/mL), which also affects the compound’s absorption and bioavailability [6]. Furthermore, its isomerization (e.g., through light exposure) is related to complications in terms of storage, application, and efficacy [7,8]. For instance, it was found that the photochemical transformation of resveratrol leads to production of isomers and derivatives, namely cis-resveratrol, 2,4,6-trihydroxy-phenanthrene, and resveratrone [9]. In this regard, the incorporation of resveratrol into nanoparticles could be considered as a suitable strategy to improve its stability and bioavailability [10,11].

The utilization of nanoparticle-based drug delivery systems in modern therapies could provide greater precision and effectiveness in the administration of drugs. Specifically, these nanocarriers have the capacity to increase the aqueous solubility and stability of resveratrol, thereby providing significant therapeutic concentrations in the plasma and improving bioavailability [12]. Nanomicelles, which consist of amphiphilic polymers, allow for a high encapsulation degree of hydrophobic molecules [13]. Pluronics are triblock copolymers, which are considered safe and biocompatible by the US Food and Drug Administration (FDA) and can be used for the preparation of micelles [14]. For instance, micelles prepared from Pluronic F127, or combination of Pluronic F127 and casein, increased the solubility of resveratrol 50 times [15]. Increased solubility was also observed after loading resveratrol in poly(methacrylic acid)-b-poly(ε-caprolactone)-b-poly(methacrylic acid) micelles [16], while enhanced photostability was registered after encapsulation in Soluplus copolymeric micelles [17]. Polymeric nanoparticles prepared with natural polymers could also overcome the disadvantages of resveratrol [18]. For instance, there was a 10-fold increase in the oral bioavailability of resveratrol after encapsulation in casein nanoparticles [19].

Albumin has also been extensively examined as a drug carrier, due to its biodegradability, low toxicity, non-immunogenicity, and biocompatibility [20,21]. Hydrophobic drugs, such as resveratrol, can be encapsulated into albumin nanoparticles owing to the presence of non-polar protein residues, forming hydrophobic domains. In addition, an interaction between hydroxyl groups of resveratrol and oxygen atoms of albumin is also possible [22]. It was reported that the formation of a complex between bovine serum albumin (BSA) and trans-resveratrol increased its solubility and stability [23]. The thermal and UV light stability of resveratrol, as well as its antioxidant activity, were enhanced after loading in zein, zein–albumin and zein–albumin–caffeic acid nanoparticles [24].

The purpose of our research was to demonstrate the advantages of encapsulation of resveratrol in nanosized drug delivery systems, namely mixed Pluronic F127 and P123 micelles, and bovine serum albumin nanospheres. The obtained nanosized delivery systems were characterized with respect to their encapsulation efficiency, size, zeta potential, XRD, and in vitro release. The potential of the carriers to enhance the photostability of resveratrol under daylight conditions and UV irradiation was also evaluated. Moreover, the antioxidant capacity of the systems was examined via ABTS assay, as well as in an in vitro model of oxidative stress in L929 fibroblasts.

## 2. Results and Discussion

### 2.1. Development and Characterization of Resveratrol-Loaded Micelles and Nanospheres

**Figure 1:**
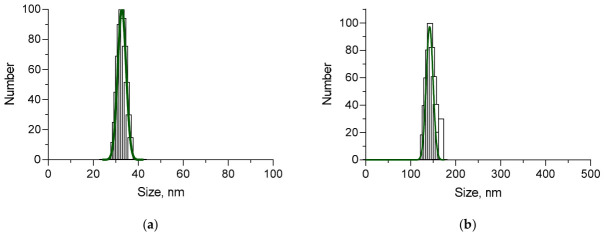
Histograms depicting the mean diameters (measured via DLS) of resveratrol-loaded Pluronic micelles (**a**) and albumin nanospheres (**b**).

**Figure 2:**
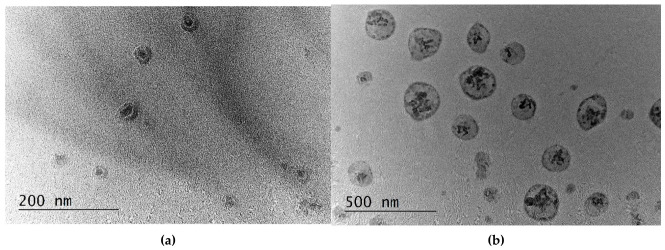
TEM photographs of resveratrol-loaded micelles (**a**) and albumin nanospheres (**b**).

Trans-resveratrol was successfully loaded in Pluronic micelles and bovine serum albumin nanospheres through film hydration and desolvation methods, respectively. This led to the production of nanoparticles with different characteristics. In particular, the loaded micelles possessed a mean diameter of approx. 33 nm (Figure 1a), narrow size distribution, and zeta potential of −4.0 mV (Table 1). Despite the low absolute value of the zeta potential, the micelles are considered physically stable structures, owing to steric stabilization by the polyethylene oxide (PEO) corona. The albumin nanospheres possessed larger size than the micelles, approx. 145 nm (Figure 1b), low polydispersity index of 0.251, and negative zeta potential (−22.6 mV) (Table 1). Such characteristics are typical for albumin nanoparticles. For example, Abolhassani and Shojaosadati observed mean diameters of 170.7 and 187.3 nm, and zeta potentials of −31.2 and −23.8 mV, for piperine-loaded human albumin nanoparticles obtained by desolvation and self-assembly techniques, respectively [25]. Tamoxifen-loaded human serum albumin nanoparticles produced by desolvation method were characterized with 195 nm mean size and −21 mV zeta potential [26]. The transmission electron microscopy (TEM) images confirmed the size difference between the two types of resveratrol-loaded polymeric systems (Figure 2). The possible reason for the larger size of albumin nanoparticles observed by TEM is the aggregation that could occur during the drying stage in the sample preparation. Nevertheless, the sizes of both nanosystems were below 200 nm, which is a key factor for the avoidance of macrophage phagocytosis and increase in circulation time [27].

The encapsulation efficiency for resveratrol was higher in the micelles (more than 89%) compared to the albumin nanospheres (76.2%) (Table 1). The same tendency was observed for the drug loading content, namely 10.22% in the micelles vs. 1.77% in the albumin particles. This can be explained with the different structures of the nanoparticles and the characteristics of the polymers used. In our previous study, we observed a very high affinity of resveratrol to the polypropylene oxide (PPO) chains (13.35 for the Flory–Huggins parameter), which indicated its prevalent encapsulation in the micellar core. This was confirmed via X-ray diffraction (XRD) analysis [28]. Regarding the albumin nanoparticles, the protein is highly hydrophilic, which decreases the affinity of resveratrol. The calculated Flory–Huggins parameter for albumin was found to be 54.14, which indicated a much lower affinity of resveratrol to the protein compared to the PPO core of the micelles (Table 1). Nevertheless, the presence of hydrophobic domains in the protein structure allowed for a sufficient loading of hydrophobic polyphenols [22]. Thus, the high encapsulation efficiency of resveratrol in albumin nanoparticles in our study was probably due to the inclusion of resveratrol in such hydrophobic domains.

**Figure 3:**
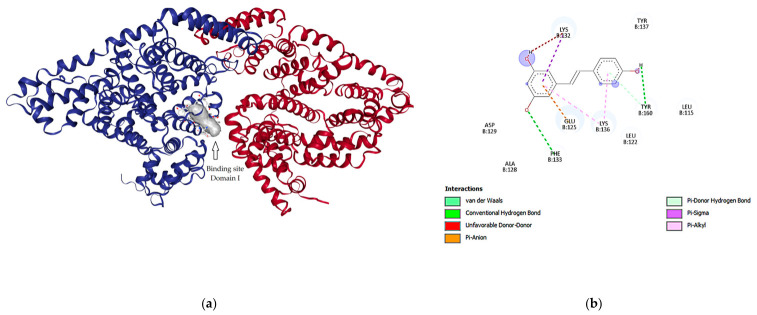
Three-dimensional presentation of the molecular docking obtained via CB-Dock2 (**a**), and two-dimensional presentation of the interactions between resveratrol and albumin revealed via BIOVIA Discovery Studio Visualizer (**b**). Abbreviations: LYS—lysine, PHE—phenylalanine, TYR—tyrosine, GLU—glutamic acid.

**Figure 4:**
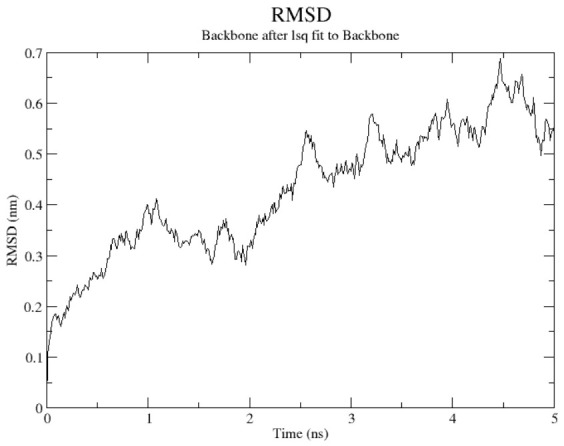
RMSD values (nm) vs. time (ns) during the simulation of resveratrol–albumin complex.

In order to gain an insight into the interactions between resveratrol and albumin in the nanospheres, molecular docking (Figure 3a) and dynamics (Figure 4) were conducted. Table 2 presents the binding energies and the corresponding cavity volumes. It was found that the interactions between resveratrol and albumin for the conformation with the highest binding activity (Vina Score of −7.8) were due to hydrogen bonds and π-interactions with the following amino acid residues: LYS B:136 (π-alkyl), PHE B:133 (conventional hydrogen bond), TYR B:160 (conventional and π-donor hydrogen bond), GLU B:125 (π-anion), and LYS B:132 (π-sigma). The presence of such non-covalent and reversible bonds could explain the lower encapsulation efficiency in the case of the albumin nanospheres compared to the micelles.

**Figure 5:**
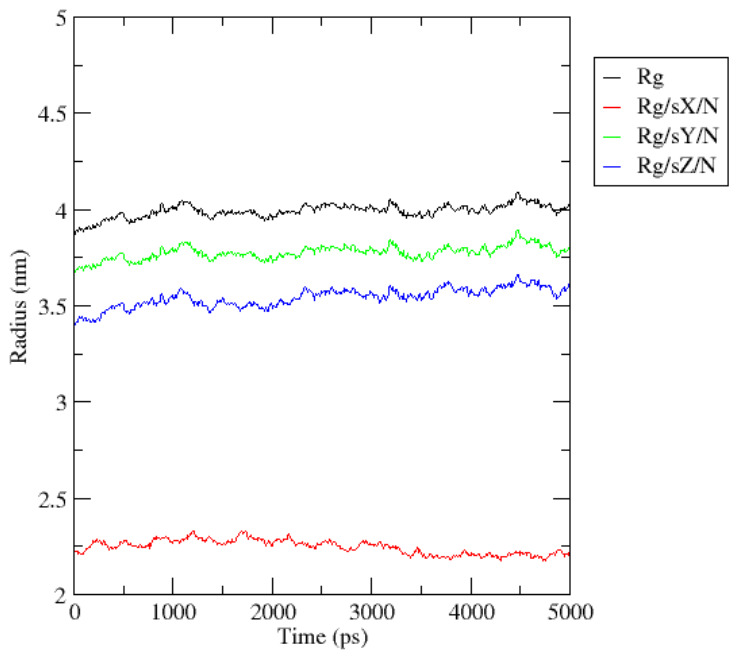
Radius of gyration (Rg) of the resveratrol–albumin complex.

The RMSD (root mean square deviation) data (Figure 4) show the conformational changes in the protein over time, which is important for the overall stability of the complex. In our study, values between 0.3 and 0.7 nm after the initial rise were observed. The smaller the variation, the more stable the complex. Another important parameter is the radius of gyration that indicates the compactness of the system over time. According to the radius of the gyration data extracted from our simulation test, there was a stable overall value, without large fluctuations (Figure 5). Therefore, despite the possible changes in the conformation of the complex over time, the size and compactness are stable, which is an important factor for the physical stability of the nanoparticles.

**Figure 6:**
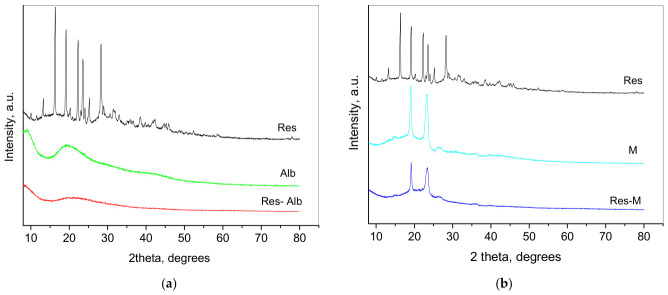
XRD patterns of resveratrol loaded in albumin nanospheres (**a**) and micelles (**b**). The abbreviations are as follows: Res—free resveratrol, Alb—empty albumin nanospheres, Res-Alb—resveratrol-loaded albumin nanospheres, M—empty Pluronic micelles, and Res-M—resveratrol-loaded micelles.

Furthermore, XRD analyses were performed (Figure 6). Pure resveratrol exhibited a highly crystalline nature. The diffraction peaks corresponded to the trans form of resveratrol, as previously reported by Caruso et al. [29]. The compound crystallizes in the P2_1_/c space group, with refined unit cell parameters a = 4.463(7) Å, b = 9.235(2) Å, c = 26.682(4) Å, and β = 92.64(2)°. The mean crystallite size was 53 nm. In contrast, the X-ray diffraction pattern of albumin displays three broad amorphous halos centered at 9.02°, 19.38°, and 42.37° (2θ). For the resveratrol–albumin (Res-Alb) sample, the diffraction data indicated a loss of crystallinity of resveratrol, probably due to a molecular dispersion within the carrier, rather than a reduced drug concentration below XRD detection limits. The latter is confirmed by the high values of drug loading content. The disappearance of the peaks at 9.02° and 42.37° of the albumin for the loaded sample, together with the shift of the main amorphous halo of albumin from 19.38° to 21.4° (2θ), suggested a contraction of internal bonding distances due to structural rearrangements following resveratrol incorporation.

The micellar system exhibited both crystalline and amorphous characteristics, wherein the crystalline contribution arises from the PEO segments, while the PPO blocks account for the amorphous portion, classifying the structure as semicrystalline [30]. The percentage crystallinity represents the proportion of crystalline material relative to the total material present. In this study, empty micelles (M) showed a crystallinity of 32%, reflecting the combination of Pluronic F127 (54% crystallinity) and Pluronic P123 (12% crystallinity). Upon loading of resveratrol into the micelles, the overall crystallinity decreased to 21%. Moreover, the incorporation of resveratrol led to a complete deterioration of its crystalline structure, as no characteristic diffraction peaks of resveratrol were detected in the XRD patterns of the loaded micelles (Res-M).

### 2.2. In Vitro Release Studies

**Figure 7:**
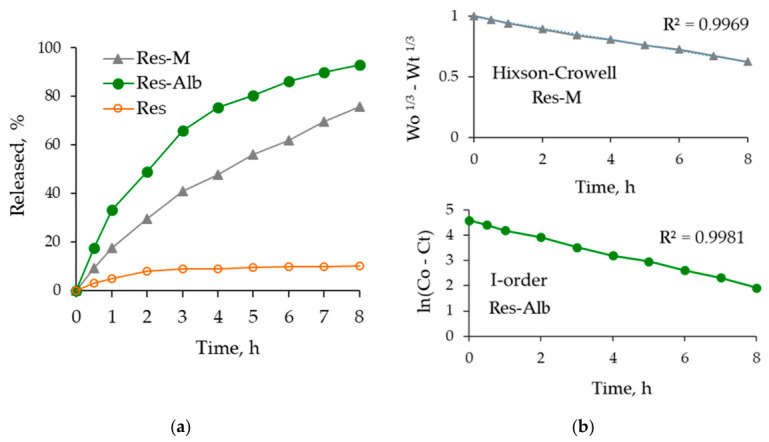
In vitro dissolution profiles of pure resveratrol (Res), resveratrol loaded in micelles (Res-M) or albumin nanospheres (Res-Alb) (**a**), and fit of the release data to the Hixson–Crowell model (for Res-M) and first-order kinetic model (for Res-Alb) (**b**).

In order to evaluate the delivery process of resveratrol from the Pluronic micelles and albumin nanospheres, in vitro release studies were conducted. First, a significant increase in the dissolution of resveratrol was observed after encapsulation in the nanoparticles, especially in the albumin nanospheres (Figure 7). In particular, after 8 h, only around 10% of resveratrol (in suspension) was dissolved in the medium, vs. 76% released from the micelles and 93% from the albumin nanoparticles. This result was related to the loss of crystallinity of resveratrol after its loading in the nanosystems (shown in Figure 6). Furthermore, the release rate of resveratrol from the micelles was slower compared to that from the albumin nanospheres. The latter was probably due to the higher affinity of the drug to the PPO chains in the micellar core compared to its affinity to albumin (Table 1). In addition, the weak non-covalent and reversible bonds between resveratrol and albumin (shown in Figure 3b) also contributed to the faster release from the albumin nanoparticles. The mechanism of drug release was studied by fitting the data to zero-order, first-order, Higuchi, Korsmeyer–Peppas and Hixson–Crowell models (Table 3). The highest correlation coefficient (r^2^) for the resveratrol-loaded micelles was for the Hixson–Crowell model (Figure 7b), indicating a release mechanism dominated by change in particle size. Regarding the albumin nanospheres, the highest coefficient was found for first-order kinetics (Figure 7b), indicating that the release depends mainly on the remaining concentration of resveratrol within the nanoparticles.

### 2.3. Photostability Studies

**Figure 8:**
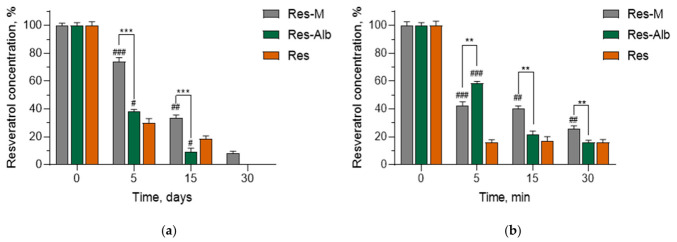
Concentrations of the non-degraded resveratrol after exposure of the free drug (Res), drug loaded in micelles (Res-M), or drug loaded in albumin nanospheres (Res-Alb) to daylight (**a**) or UV irradiation (**b**). # *p* < 0.05, ## *p* < 0.01, and ### *p* < 0.001 vs. Res; ** *p* < 0.01 and *** *p* < 0.001 between Res-M and Res-Alb.

The photostability of active substances could be considered a significant issue regarding many stages of drug handling, including the preparation, storage, transport, and application of a delivery system [31]. The encapsulation of photosensitive drugs is considered one of the most researched strategies for overcoming this issue [32]. The isomerization of trans- to cis-resveratrol is a well-known phenomenon that leads to production of a substance with hindered pharmacological activities. In this process, other byproducts have also been detected, for example, 2,4,6-trihydroxyphenanthrene (which is marked as potentially harmful and originates from the cis form) [33]. This could be a prerequisite for occurrence of toxic reactions and compromised therapy. Taking this into consideration, our next task was to evaluate the capability of the developed polymeric nanoparticles to protect resveratrol from light-induced isomerization. Two conditions were tested, namely exposure of non-loaded and encapsulated resveratrol to daylight for 30 days (Figure 8a), and UV irradiation for 30 min (Figure 8b). As can be observed, the encapsulation into the nanoparticles significantly reduced the isomerization of trans- to cis-resveratrol. Better protective effect was observed in the case of the polymeric micelles. In particular, after 5 days of daylight exposure, there was approx. 26% degradation of the polyphenol in the micelles, vs. 62% in the albumin nanospheres, and 70% for the non-loaded resveratrol. After 30 days, there was a trace of non-degraded resveratrol only in the micellar system. Similar results were observed for the UV irradiation. After 15 and 30 min, the best protective effect was observed for the micelles again, namely degradation of 60% and 74%, vs. 78% and 84% in the albumin particles, and 83% and 84% for the non-loaded resveratrol. Lopez-Hernandez et al. observed that, after irradiation of trans-resveratrol with 254 nm UV light, there was transformation of the trans form to cis form, and, after 30 min, a third compound that is generated from the cis form (having strong fluorescence) was detected [8]. In our study, we also evaluated the transformation via HPLC analysis of both types of nanoparticle dispersions exposed to daylight or UV irradiation. The results showed that, without the exposure, there was only trans-resveratrol in the chromatograms (t_R_ = 8 min, absorbance at 306 nm). However, after 5 days of daylight exposure or 15 min of UV irradiation, cis-resveratrol (t_R_ between 8.5 and 8.7 min, absorbance at 285 nm) and a third compound, which was probably produced from the cis form (t_R_ = 10 min, absorbance at 260 nm), were registered (Figures S1 and S2). The wavelengths match the ones reported by Lopez-Hernandez et al. [8]. Similarly, Montsko et al. reported production of a third compound after UV irradiation of trans-resveratrol. They discovered that this compound was derived through oxidation and change in the double bond at the center of the resveratrol molecule to a triple bond, forming a diphenylacetylene derivative (3,4′,5-trihydroxy-diphenylacetylene) [34]. Furthermore, in our study, it was well distinguished that the intensity of the peak of cis-resveratrol was higher for the non-loaded drug compared to the encapsulated drug. Table 4 presents the values from the ratio between trans- and cis-resveratrol in the dispersions after exposure to daylight (5 days) and UV irradiation (15 min). As can be seen, a higher value from the ratio was obtained for resveratrol loaded in micelles and albumin nanoparticles, confirming that they preserved the drug. Interestingly, the protection was more efficient in the case of the micelles. Nevertheless, the studies revealed the capability of the developed polymeric nanoparticles to reduce the isomerization of resveratrol, allowing for the production of stable, safe and effective formulations.

### 2.4. Antioxidant Properties

**Figure 9:**
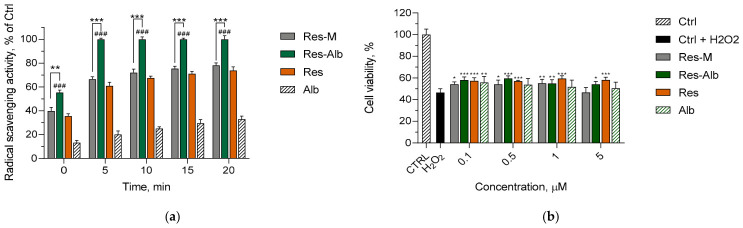
ABTS scavenging activity (**a**) and protective antioxidant activity against H_2_O_2_-induced oxidative stress in L929 fibroblasts (**b**) of free resveratrol (Res), resveratrol loaded in micelles (Res-M), loaded in albumin nanospheres (Res-Alb), and empty albumin nanospheres (Alb). ### *p* < 0.001 vs. Res (for ABTS test); * *p* < 0.05, ** *p* < 0.01 and *** *p* < 0.001 between Res-M and Res-Alb (for ABTS test) or vs. H_2_O_2_-treated group for the cell model.

The antioxidant properties of trans-resveratrol are well-researched and confirmed. However, sometimes encapsulation in nanoparticles could modify the effects of antioxidants. Taking this into consideration, we evaluated the antioxidant potential of resveratrol-loaded micelles and albumin nanospheres applying ABTS testing (Figure 9a). The loading of resveratrol in micelles retained the antioxidant activity of resveratrol. In comparison, the loaded albumin nanoparticles showed significantly higher radical scavenging activity. Particularly, after 20 min of incubation, scavenging activity levels of 100%, 78%, and 74% were observed for resveratrol loaded in albumin nanospheres, loaded in micelles, and the pure drug, respectively. The empty micelles did not show any radical scavenging activity compared to the empty albumin nanospheres, which exerted approx. 33% activity after 20 min of incubation. In our opinion, the superior effect of the albumin nanoparticles could be related to the activity of albumin itself. In particular, it has been reported that bovine serum albumin possesses significant activity against the ABTS radical, due to the presence of the amino acid tyrosine [35].

The next step was to evaluate the antioxidant effects of encapsulated resveratrol against H_2_O_2_-induced oxidative stress in L929 fibroblasts (Figure 9b). First, the empty nanoparticles were found to be non-toxic to the cells. The non-encapsulated resveratrol showed statistically significant protection in all tested concentrations. At 0.1, 0.5, and 1 µM concentrations, the protective effect was retained after loading in the nanoparticles, despite the sustained release of the polyphenol. At a concentration of 5 µM, only the loaded albumin nanoparticles showed a statistically significant effect. Taking into consideration the potential of albumin to scavenge the ABTS radical, we also evaluated the protective effect of the empty albumin nanoparticles on the cells. Protection was observed only at the lowest concentration. The mechanism of albumin’s antioxidant effect includes neutralization of free radicals such as hydroxyl, peroxyl, superoxide radicals, and singlet oxygen, as well as inhibition of NADPH oxidase [36]. The micelles did not show any antioxidant activity.

## 3. Materials and Methods

### 3.1. Materials

Trans-resveratrol, bovine serum albumin (fraction V), Dulbecco’s Modified Eagle Medium, fetal bovine serum (FBS), L-glutamine, 3-(4,5-dimethylthiazol-2-yl)-2,5-diphenyltetrazolium bromide (MTT), and 2,2′-azino-bis(3-ethylbenzothiazoline-6-sulfonic acid) (ABTS) were purchased from Sigma-Aldrich (Merck KGaA, Darmstadt, Germany). Pluronic^®^ P 123 (PEO_20_PPO_70_PEO_20_) and Pluronic^®^ F 127 (PEO_101_PPO_56_PEO_101_) were obtained from BASF (Ludwigshafen, Germany). The murine fibroblast cell line L929 was gained from the European Collection of Cell Cultures (ECACC, Salisbury, UK).

### 3.2. Preparation of Resveratrol-Loaded Nanosized Drug Delivery Systems

Trans-resveratrol was loaded in two types of polymeric systems, namely micelles and nanospheres. For the preparation of the loaded micelles, 4 mg of resveratrol, as well as Pluronic P123 and Pluronic F127, at 20 mg each, were dissolved in 4 mL of methanol. Then the mixture was left under ambient conditions until the evaporation of the solvent, and the obtained film was redispersed in 4 mL purified water. For the preparation of the loaded nanospheres, 100 mg of bovine serum albumin was dissolved in 2 mL of purified water. Then 2.4 mL acetone, containing 2 mg of trans-resveratrol, was dripped into the albumin solution. The system was stirred (700 rpm) until the full evaporation of acetone. Both systems were then filtered (Nylon filter, 0.45 µm), and the filters were rinsed with 50% ethanol. The concentration of the non-loaded resveratrol in the rinsed filtered fraction was determined via HPLC method (Thermo Scientific UltiMate Dionex 3000, Chromeleon 7.2 SR3 Systems, Thermo Fisher Scientific, Waltham, MA, USA) [37], applying the following conditions: Aquasil C18 Column (250 mm × 4.60 mm, particle size 5 μm, pore size 100 Å), methanol:water (52%:48% *v*/*v*) mobile phase, isocratic elution, injection volume of 10 µL, flow rate of 1 mL/min, retention time 8 min, column temperature of 25 °C, and ultraviolet detection at 306 nm. A standard curve obtained in the range of 3–50 µg/mL (r > 0.9999) was applied for the determination of resveratrol concentration.

The encapsulation efficiency (EE) and drug loading content (DLC) were calculated according to the following equations:

(1) $$\mathrm{EE}\;(\%)\;=\;\left(\frac{C0-C1}{C0}\right)\times 100,$$

(2) $$\mathrm{DLC}\;(\%)=\left(\frac{C0-C1}{M}\right)\times 100,$$

where *C*0 is the initial amount of trans-resveratrol, *C*1 is the concentration of the non-loaded resveratrol in the rinsed filter fraction, and *M* is the weight of the nanoformulation.

### 3.3. Characterization of the Loaded Nanoparticles

The mean sizes and polydispersity of resveratrol-loaded micelles and nanospheres were determined via photon correlation spectroscopy at a scattering angle of 90° (Zetasizer NanoBrook 90Plus PALS, Brookhaven Instruments Corporation, Holtsville, NY, USA). For the evaluation of the zeta potential of the nanoparticles, the phase analysis light scattering (PALS) method, at a scattering angle of 15°, was applied. The dispersions were used without dilution.

The sizes and shapes of the loaded nanoparticles were assessed via transmission electron microscopy using an HR STEM JEOL JEM 2100 (Tokyo, Japan).

The compatibility between resveratrol and the hydrophobic chains of poly(propylene oxide) (PPO) or bovine serum albumin was evaluated via calculation of the Flory–Huggins parameter χ_sp_, applying the following equation:

Χ_sp_ = [V_s_(δ_s_ − δ_p_)^2^]/RV (3)

where V_s_ is the molar volume of resveratrol, δ_s_ and δ_p_ are the solubility parameters of resveratrol and the PPO/albumin, R is the gas constant, and T is the Kelvin temperature [38]. The solubility parameters of resveratrol and PPO block were calculated using the contributions of the chemical groups in the molecules to their cohesive energy [39].

Powder X-ray diffraction patterns of trans-resveratrol, empty micelles and nanospheres, and resveratrol-loaded nanoparticles were recorded in the 5–80° 2θ range with a step of 0.02°, applying a Bruker D8 Advance diffractometer (Bruker Corporation, Billerica, MA, USA) with Cu Kα radiation and a LynxEye detector. The degree of crystallinity (Xc) was determined from the X-ray diffraction (XRD) pattern by comparing the intensity of the crystalline peaks (*Ic*) with the intensity of the amorphous background (*Ia*), using the following equation (TOPAS V4.2) [40]:

(4) $$\mathrm{Xc}\;(\%)\;=\;\left(\frac{Ic}{Ic+Ia}\right)\times 100$$

### 3.4. Molecular Docking

The protein–ligand docking method, applying CB-Dock2 [41] (https://cadd.labshare.cn/cb-dock2/index.php, accessed on 28 November 2025), which is based on AutoDock Vina (version 1.2.0) docking [42,43,44,45], was used for the evaluation of the interactions between resveratrol and albumin. Resveratrol (CID 445154, PubChem) was used as a ligand, and bovine serum albumin (4F5S, Protein Data Bank (PDB)) [46] was used as a receptor. 2D visualization of the receptor–ligand interactions between resveratrol and albumin was prepared with BIOVIA Discovery Studio Visualizer (Dassault Systèmes, version 25.1.0.24284). Moreover, the stability of the formulated resveratrol–albumin complex was evaluated via the platform Visual Dynamics (a WEB application for molecular dynamics simulation using GROMACS; version 2025.1) [47]. The following parameters were selected: force field—AMBER99SB; water model—SPC simple point charge; box type—cubic. The resveratrol–albumin complex (.pdb) was obtained from CB-Dock2. The UCSF ChimeraX software (version 1.11rc202511271917) [48] was applied for the separation of the molecule of the complex (to ligand and receptor in the specific conformation). Thereafter, hydrogen atoms were added to the molecule of resveratrol. The ACPYPE Server [49] was applied to obtain the files of the ligand required for molecular dynamics.

### 3.5. In Vitro Release Studies

The dialysis method was applied for studying the in vitro release profiles of resveratrol from the polymeric nanoparticles. The tests were conducted in a shaking water bath at a temperature of 37 °C (IKA Labortechnik HS-B20, Staufen, Germany) with phosphate buffer as a buffer media (pH = 7.4, 10% ethanol). Briefly, 2 mL of the micelles, the albumin nanospheres, or aqueous suspension of free resveratrol was introduced in a dialysis membrane (6–8 kDa MWCO, Spectrum Labs, San Francisco, CA, USA), and then the membrane was placed in 40 mL of buffer; 2 mL samples were taken from the external medium at predetermined time intervals. The same amount of fresh buffer was returned to maintain the sink conditions. The concentration of the released resveratrol was determined by HPLC method, which is described in detail in Section 3.2.

The release data of the nanoparticles were fitted to zero-order (Equation (5)), first-order (Equation (6)), Higuchi (Equation (7)), Korsmeyer–Peppas (Equation (8)), or Hixson–Crowell (Equation (9)) models in order to evaluate the release kinetics:

C_t_ = C_0_ + K_0_·t, (5)

where C_t_ represents the amount of resveratrol released during time t; C_0_ is the initial concentration of resveratrol; and K_0_ is the zero-order rate constant.

ln(C_0_ − C_t_) = ln(C_0_) − K_1_·t, (6)

where C_t_ represents the amount of resveratrol released during time t; C_0_ is the initial concentration of resveratrol; and K_1_ is the first-order rate constant.

C_t_ = K_H_·t^1/2^, (7)

where C_t_ is the amount of resveratrol during time t, and K_H_ is the release constant of Higuchi.

M_t_/M_∞_ = k·t^n^, (8)

where M_t_/M_∞_ is the accumulated fraction of resveratrol release, t is the respective time, and k is the release rate constant.

(9) $${W_{0}}^{1/3}-{W_{t}}^{1/3}=K_{\mathrm{HC}}\cdot t$$

where W_0_ is the initial amount of resveratrol in the nanoparticles at time 0, W_t_ is the amount of resveratrol remaining in the nanoparticles at time t, and K_HC_ is the Hixson–Crowell release constant.

### 3.6. Photostability Studies

Photostability studies were conducted with free and loaded micelles or nanospheres of trans-resveratrol under daylight conditions (for 30 days) or UV irradiation (for 30 min) at room temperature. The samples (0.6 mg/mL concentration of resveratrol) were placed in glass vials. At predetermined time intervals, the concentration of non-degraded resveratrol was determined via the HPLC method, as described above. Irradiation was performed with a 400 W metal halide flood lamp (Dymax 5000-EC UV, Torrington, CT, USA) at an intensity of 23.8 mW/cm^2^ per minute.

### 3.7. ABTS Assay

The ABTS [50,51] assay was applied for the evaluation of the antioxidant potential of free (50% alcoholic solution) or loaded micelles and albumin nanospheres of resveratrol. Briefly, the ABTS radical was first activated via mixing with potassium persulfate in an aqueous solution and incubating under dark conditions at 25 °C for 16 h. Before testing, it was diluted to 5% in ethanol. Thereafter, 1 mL of each sample, containing 10 µM resveratrol, or the empty nanoparticles in corresponding concentration, was mixed with 1 mL of ABTS solution. Distilled water was used as a control sample. The absorbance was measured at 734 nm (Thermo Fisher Scientific, Waltham, MA, USA). The scavenging activity (SA) was determined by applying the following equation:

SA (%) = (A_1_ × 100)/A_0_ (10)

where A_1_ is the absorbance at each time of incubation, and A_0_ is the absorbance of the control at 0 min.

### 3.8. In Vitro Protective Effect

The hydrogen peroxide (H_2_O_2_)-induced model of oxidative stress on murine L929 fibroblasts was applied for evaluation of the protective antioxidant potential of pure or loaded resveratrol. The L929 cells were maintained in Dulbecco’s Modified Eagle Medium (low glucose), supplemented with 10% fetal bovine serum and 4 mM L-glutamine. They were seeded in 96-well plates at a cell density of 2 × 10^4^ and incubated overnight at standard conditions of 37 °C, 5% CO_2_, and high humidity (Esco CelCulture^®^ CO_2_ Incubator, CCL-170B-8-IVF, Esco Micro Pte. Ltd., Singapore). After 24 h of incubation, the fibroblasts were treated for 2 h with resveratrol-loaded micelles, resveratrol-loaded albumin nanospheres, free resveratrol (in ethanol stock solution) at 0.1, 0.5, 1, and 5 µM concentrations, or the empty nanoparticles in corresponding concentrations. Then, 500 µM H_2_O_2_ in PBS (containing Ca^2+^ and Mg^2+^) was applied to the cells for 1h. Thereafter, each well was washed with PBS (containing Ca^2+^ and Mg^2+^), and fresh medium was added. After incubation for 24h, an MTT solution in PBS (10 mg/mL) was added to each well. The plates were incubated at 37 °C for 3h. After that, the MTT solution was carefully aspirated, and 100 μL of DMSO was added to each well in order to dissolve the formed formazan crystals. The absorbance was measured in a multiplate reader, Synergy 2 (BioTek Instruments, Inc., Highland Park, Winooski, VT, USA) at 570 nm (690 nm for background absorbance).

### 3.9. Statistical Analysis

All experiments were conducted in triplicate, and the results are expressed as mean values ± SD. GraphPad Prism 8 Software (Dotmatics, San Diego, CA, USA) was applied for performing the statistical analyses. One-way ANOVA with Dunnett’s multiple comparison post-test was applied for the comparison with the H_2_O_2_-treated group in the in vitro cell experiment. Multiple *t*-tests with Holm–Sidak correction were applied for comparing Res-Alb and Res-M in the photostability and antioxidant studies. *p* < 0.05 was considered significant.

## 4. Conclusions

In the current study, encapsulation of resveratrol in two types of polymeric nanoparticles, namely Pluronic micelles and albumin nanospheres, was applied as a strategy for coping with some of the problematic issues of the drug. The developed nanoparticles were characterized by mean diameter smaller than 200 nm, high encapsulation efficiency and improved dissolution. Importantly, the photostability of resveratrol under daylight exposure and UV irradiation was significantly increased, especially after encapsulation in the Pluronic micelles. The antioxidant effects (ABTS scavenging activity and in vitro protective effect in L929 fibroblasts) were also enhanced, although a more significant effect was exerted by the albumin nanospheres. Therefore, this study revealed the opportunity to stabilize the drug against photodegradation and enhance its antioxidant activity, which could expand its therapeutic application.

## Figures and Tables

**Table 1:** Mean size, polydispersity index (PDI), zeta potential, encapsulation efficiency (EE), and values for Flory–Huggins parameter of resveratrol-loaded micelles (Res-M) and albumin nanospheres (Res-Alb).

Sample	Mean Size, nm	PDI	Zeta Potential, mV	EE, %	Flory–Huggins Parameter
Res-M	32.76 ± 1.9	0.278 ± 0.015	−4.0 ± 1.8	89.4 ± 7.7	13.35
Res-Alb	144.6 ± 7.5	0.251 ± 0.018	−22.6 ± 1.1	76.2 ± 11.4	54.14

**Table 2:** Binding energy levels and related cavity sizes for resveratrol and albumin.

Vina Score	Cavity Volume (Å3)	Center (x, y, z)	Docking Size (x, y, z)
−7.8	4101	49, 22, 93	35, 21, 21
−7.3	8389	0, 26, 108	35, 35, 27
−7.2	3494	20, 24, 101	28, 31, 35
−7.0	8957	68, 22, 85	35, 35, 29
−6.5	18,855	47, 22, 119	35, 35, 21

**Table 3:** Kinetic parameters for the release data fitted to zero-order, first-order, Higuchi, Korsmeyer–Peppas and Hixson–Crowell models.

Sample	0-Order	I-Order	Higuchi	Korsmeyer–Peppas	Hixson–Crowell
Res-M	0.9734	0.9955	0.9828	0.9950 *n* = 0.7891	0.9969
Res-Alb	0.8852	0.9981	0.9836	0.9798 *n* = 0.7408	0.9807

**Table 4:** Calculated values from the ratio of trans- to cis-resveratrol in the samples exposed to daylight (5 days) or UV irradiation (15 min).

Sample	Daylight Exposure	UV Irradiation
Res	0.899	0.648
Res-Alb	1.397	0.991
Res-M	10.74	2.301

## Data Availability

The original contributions presented in this study are included in the article/Supplementary Materials. Further inquiries can be directed to the corresponding author.

## Supplementary Materials

The following supporting information can be downloaded at: https://www.mdpi.com/article/10.3390/ijms27135846/s1.
