# Supplementary material for: Improving the Photostability and Antioxidant Activity of Resveratrol via Incorporation in Two Types of Polymeric Nanoparticles
Source: Int J Mol Sci. 2026 Jun 29;27(13):5846. doi: 10.3390/ijms27135846 (PMC13362059; doi:10.3390/ijms27135846)
Supplement: Supplementary file 1 [file ijms-27-05846-s001.zip › ijms-4325704-supplementary.pdf]

Improving the Photostability and Antioxidant Activity of Resveratrol via Incorporation in Two Types of Polymeric Nanoparticles

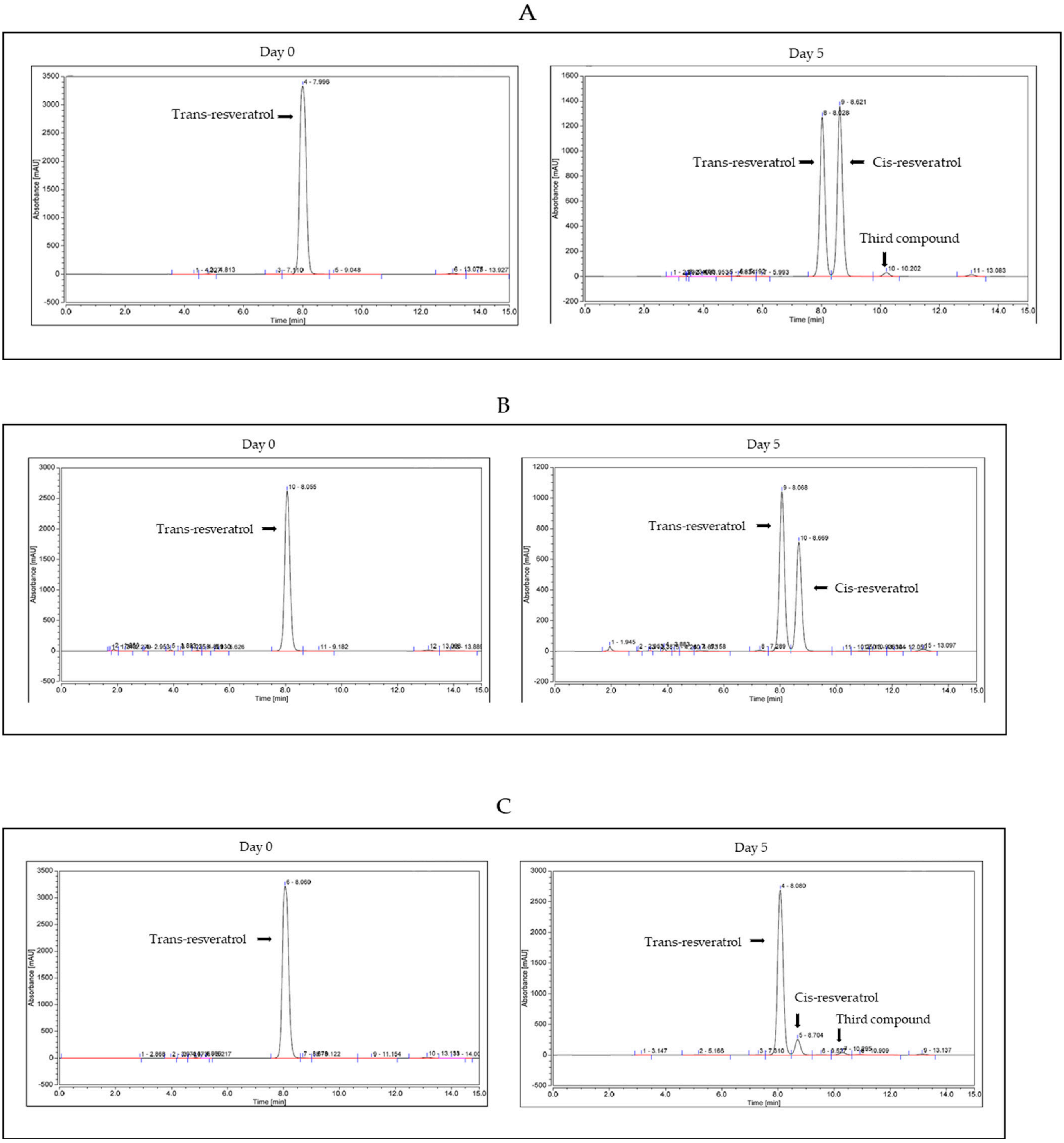

Figure S1. Chromatograms of free resveratrol (A), loaded in the albumin nanospheres (B) or in the micelles (C) without exposure (Day 0) and after exposure to daylight (Day 5).

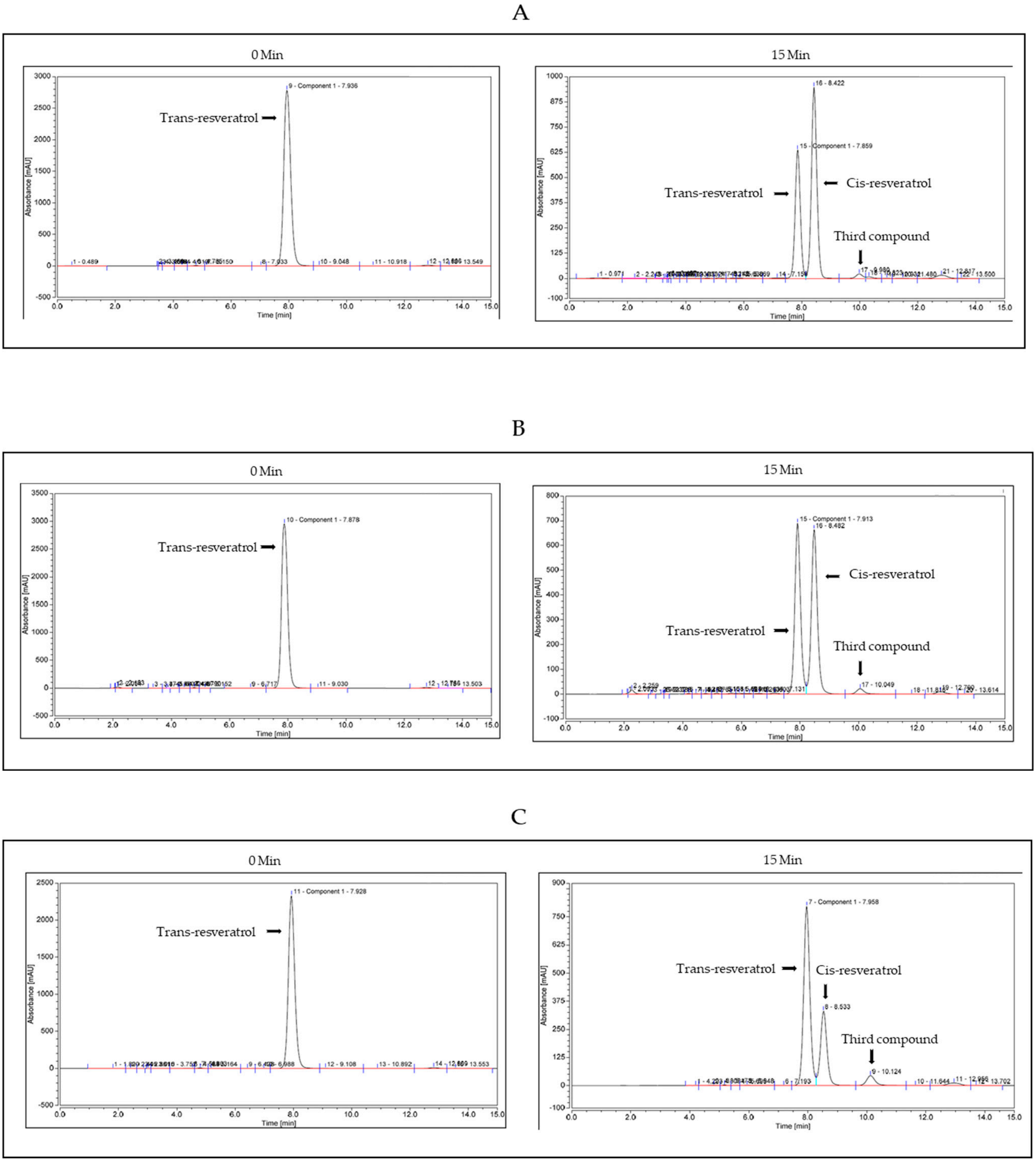

Figure S2. Chromatograms of free resveratrol (A), resveratrol loaded in the albumin nanospheres (B) or in the micelles (C) without UV-irradiation (0 min) and after UV-irradiation (15 min).
